# Genome Sequence of a *Salmonella* Phage Used To Control *Salmonella* Transmission in Swine

**DOI:** 10.1128/genomeA.00521-14

**Published:** 2014-09-11

**Authors:** Jiayi Zhang, Yingying Hong, Nicholas J. Harman, Archana Das, Paul D. Ebner

**Affiliations:** Department of Animal Sciences, Purdue University, West Lafayette, Indiana, USA

## Abstract

*Salmonella* shedding in swine often increases in response to transportation and lairage. We previously demonstrated that such increases can be limited by directly feeding microencapsulated *Salmonella* bacteriophages. Here we present the genome sequence of vB_SalM_SJ_3, a broader spectrum Viuna-like *Salmonella* phage used in those studies.

## GENOME ANNOUNCEMENT

Previously, we demonstrated that *Salmonella* shedding and transmission in swine could be controlled or limited by direct feeding of microencapsulated bacteriophages to *Salmonella* colonized pigs (1, 2, 3). Here we present the genome sequence of one lytic *Salmonella* phage with a broader host-range used in those studies.

Phage DNA was purified from a PEG 8000-precipitated lysate and sequenced by pyrosequencing (454, Eurofins MWG Operon, Huntsville, AL) and sequence reads were assembled *de novo* using Newbler (version 2.6) and DNASTAR (Madison, WI). Sequencing yielded a total of 18,975 reads with an average read length of 586 bp and average coverage of 68.29. Putative coding sequences (CDSs) were predicted using Glimmer 3.0 (4) and putative tRNAs were predicted using both tRNAscan-SE 1.21 (5) and ARAGORN (6). The genome was annotated using BLASTp searches for homology in the non-redundant protein sequences database in GenBank (7).

The vB_SalM_SJ_3 genome consisted of 162,910 bp with a G+C content of 44.38%. The genome contained 214 predicted CDSs. Of those CDSs, 73 matched identified phage genes, including 24 genes involved in phage structure, 21 genes involved in DNA replication, 13 genes involved in phage physiology, 9 genes involved in nucleotide biosynthesis, 4 regulatory genes, and 2 homing endonucleases. The remaining 141 CDSs encoded hypothetical proteins. No genes associated with lysogeny, toxin production, *Salmonella* virulence, or antibiotic resistance were identified. Further analysis, however, will be required to fully characterize and assign potential functions to the numerous unidentifiable or hypothetical genes.

The genome of vB_SalM_SJ_3 showed approximately 90% nucleotide homology to *Salmonella* phage Vi01 and other Viuna-like phages, and therefore was classified as a Viuna-like phage. The vB_SalM_SJ_3 major head capsid protein was compared to those of seven other phages classified as Viuna-like phages according to the International Committee on Virus Taxonomy (http://www.ictvonline.org; accession no. JN126049, JN673056, NC_016570, HQ259103, NC_019925, NC_015296, NC_013693) using clustalW. The major capsid protein of vB_SalM_SJ_3 differed by only one residue with that of *Salmonella* phage SF10. It was most distantly related to the major capsid proteins of *Shigella* phage Ag3 and *Dickeya* phage limestone, differing by 3.3 residue substitutions per 100 residues. Several unique CDSs were identified in the vB_SalM_SJ_3 sequence, however, during whole genome alignment. These included a CDS from 33,249 to 35,375 coding for a tailspike protein which aligned to *Salmonella* phage PhiSH19 with 99% coverage, but with only 51% identity, a CDS from 73,875 to 74,825, which aligned to only a part of a putative homing endonuclease of *Dickeya* phage limestone, and two CDSs from 38,925 to 40,841 and 74,822 to 75,115, which did not match any existing proteins in the NCBI non-redundant protein database (8).

### Nucleotide sequence accession number.

The complete sequence of vB_SalM_SJ3 was deposited in GenBank under the accession no. KJ174318.
